# First record of the twostripe goby, *Valenciennea helsdingenii* (Gobiidae, Gobiiformes) from the southeast coast of India

**DOI:** 10.3897/zookeys.323.5440

**Published:** 2013-08-14

**Authors:** K. Kannan, K. Sureshkumar, L. Ranjith, K. K. Joshi, M. S. Madan, Sajan John

**Affiliations:** 1 Tuticorin Research Centre of CMFRI, Tuticorin - 628 001, Tamilnadu, India; 2 Central Marine Fisheries Research Institute, Kochi - 682 018, Kerala, India; 3 Dakshin Foundation, 88/3, Sahakaranagar “A” Block, Bangalore, India

**Keywords:** Gobiidae, Bay of Bengal, Tuticorin, geographical range, Gulf of Mannar

## Abstract

Two specimens of *Valenciennea helsdingenii* (Bleeker, 1858) were collected off Punnakayal coast, from Gulf of Mannar, southeast coast of India in November 2012. The morphometric and meristic characters of the recorded specimens are described and discussed. This is the first record of the species from the Indian waters that is a range extension of its known range within the Indian Ocean.

## Introduction

The Gobiidae constitute one of the largest families of percomorph fishes. The family has a total of over 1,640 species belonging to six subfamilies (Pezold 1993, Hoese and Larson 1994, Nelson 2006). These subfamilies are distributed in reef environments of the Indian and Pacific oceans, which are home to the greatest diversity of gobiid fishes. Recent re-evalutaion of gobioid systematic using molecular methods resulted in six family clade-based classification for the family Gobiidae that includes all the genera of the former subfamilies (Thacker 2003, 2009, 2011, Ruber and Agorreta 2011). The gobiine genus *Valenciennea* has 15 recognised species, including *Valenciennea helsdingenii* that is one of larger sized species with the documented maximal total length of 25 cm (Kuiter 1993). Prior to the discovery of the material reported on in this paper, the known range of *Valenciennea helsdingenii* included the Marquesas Islands, Japan, the Philippines, Indonesia, New Britain, the Solomon Islands, the Great Barrier Reef and New South Wales, Australia, Saudi Arabia, Maldives, Seychelles, and southern Africa (Hoese and Larson 1994, Lieske and Myers 1994, Clark et al. 2000, Randall et al. 1990, Randall et al. 1997). In India, about 150 species of gobiids have been reported (Day 1876, Jones and Kumaran 1980, Murty 2002) but the finding of *Valenciennea helsdingenii* represents the first occurrence of the species from the southeast coast of India and an extension of its range within the Indian Ocean.

## Material and methods

**Figure 1.**
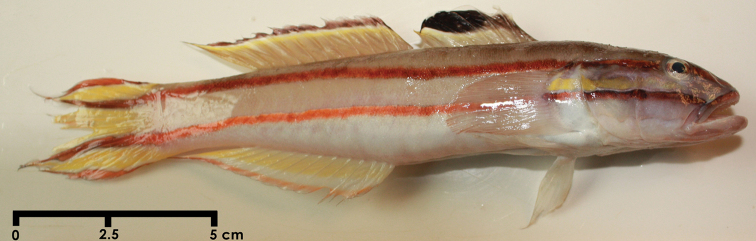
*Valenciennea helsdingenii*, 145 mm SL from the Gulf of Mannar, southeast coast of India.

**Figure 2.**
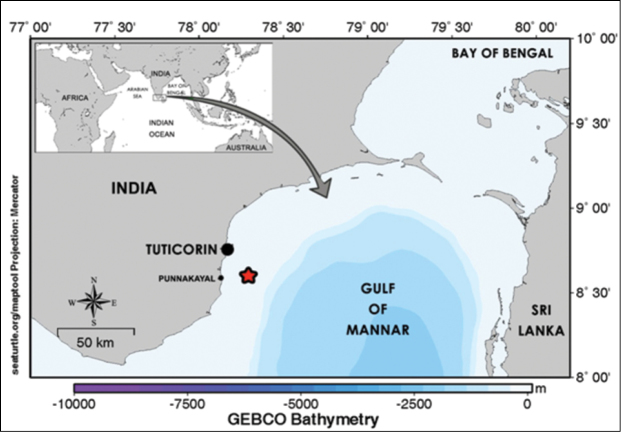
Capture location of *Valenciennea helsdingenii* (red star) in the Gulf of Mannar, southeast coast of India.

Two specimens of *Valenciennea helsdingenii* (Bleeker, 1858) (Fig. 1) were collected from the Punnakayal fish landing centre located about 15 km south of major port town of Tuticorin on 16 November 2012. The capture location was in the Gulf of Mannar (8°38'127"N, 78°12'612"E), 20 km southeast of Tuticorin (Fig. 2) at a depth of 30 to 50 m by a drift gill net operated from traditional fishing craft. The specimens were preserved in 5% formalin and brought to the laboratory for a detailed examination. Morphometric measurements were taken to the nearest millimeter using digital calipers according to Hubbs and Lagler (1958). The specimens are deposited in the National Marine Biodiversity Referral Museum at the Central Marine Fisheries Research Institute, Cochin.

## Results

### Valenciennea helsdingenii

(Bleeker, 1858)

http://species-id.net/wiki/Valenciennea_helsdingenii

#### Material examined.

Two specimens of *Valenciennea helsdingenii* (Bleeker, 1858) of SL 97 mm (GB.31.66.230.1) and 145 mm (GB.31.66.230.1.1) were deposited in the Designated National Repository, Central Marine Fisheries Research Institute, Cochin, India.

#### Description.

The body is elongate and compressed, the tongue adnate, the head is slightly compressed.The pelvic fins are completely separated, no membrane is present between the first and the second dorsal fins. The first dorsal fin is shallow and its margin rounded, the fourth spine is slightly longer than the other spines, and the caudal fin is deeply emarginate. The specimens have a pair of elongated caudal fin filaments that makes the caudal fin a peculiar shape as typical for the species. Specimens above 70 mm SL show the presence of the elongated caudal fin filaments (Hoese and Larson 1994). Body is covered with ctenoid scales while anteriorly under the middle of the first dorsal fin and on the belly th scale are cycloid; sides of the nape and the pectoral base are scaled; the prepelvic area are naked; the longitudinal-scale count is 142; the transverse-scale count is 40.

**Colour.** Overall colouration is similar to that described by Hoese and Larson (1994). The top of the head and the dorsal surface of the body are brownish gray; the rest of the head and the body is white to pale gray.

The body has two dark red stripes, the dorsal stripe extending from the front of the snout through the eye and just above the pectoral base and along the upper body to the tip of the upper caudal filament; the ventral stripe runs parallel to the first stripe, beginning at the side of the upper lip, extending across the upper part of the preoperculum and middle of the operculum, over middle of the pectoral base, continuing on the body behind the pectoral base, and reaching the tip of the lower caudal filament.The colour of the stripes is dark red to reddish brown, darkest anteriorly, and the stripes on the caudal filaments are outlined in white. The eye is yellowish white dorsally and ventrally with a reddish brown stripe through the middle, and the lower lip is white. The first dorsal fin possesses a large oval black spot extending between the third and the fifth dorsal spines.

**Table 1.** Morphometric and meristic characters of *Valenciennea helsdingenii* from the Gulf of Mannar, southeast coast of India.

**Morphometric measurements**	**GB.31.66.230.1**		**GB.31.66.230.1.1**	
	**mm**	**% SL**	**mm**	**% SL**
Standard length (SL)	97	−	145	−
Head length (HL)	23	23.7	37	25.5
Eye diameter	4	4.1	5	3.4
Postorbital length	12	12.4	19	13.1
Upper jaw length	10	10.3	16	11.0
Lower jaw length	9	9.3	15	10.3
Preorbital length	9	9.3	14	9.7
Predorsal length	32	33.0	48	33.1
Prepectoral length	27	27.8	45	31.0
Prepelvic length	27	27.8	42	29.0
Preanal length	56	57.7	86	59.3
Body depth (max.)	17	17.5	26	17.9
Caudal peduncle length	16	16.5	25	17.2
Caudal peduncle width	11	11.3	15	10.3
Distance between anal fin and anus	2	2.1	4	2.8
Distance between pelvic fin and anal fin	29	29.9	45	31.0
**Fin-ray counts**				
First dorsal	VI		VI	
Second dorsal	I11		I12	
Pectoral	22		22	
Pelvic	6		6	
Anal	I11		I12	
Segmented caudal	17		17	
Branched caudal	13		13	

#### Remarks.

*Valenciennea helsdingenii* is easily distinguished from other species of the genus in having two dark red stripes from the snout to the tip of the caudal fin, stripes on the caudal-fin filaments outlined in white and the presence of filamentous caudal rays in adults. The species was first described as *Eleotriodes helsdingenii* by Bleeker (1858), based on specimens collected from Pulau-Pulaus Gorong, Indonesia.

## Discussion

Hoese and Larson (1994) revised Indo-Pacific gobiid fishes and described seven new species from this area. Among these species, *Valenciennea helsdingenii* shows wide distribution from Southern Red sea, east Africa to Indonesia and Japan to the Great Barrier Reef (Clark et al. 2000, Lieske and Myers 1994, Randall et al. 1990). *Valenciennea sexguttata* (Valenciennes, 1837) was distributed along the Red sea, Persian Gulf, East Africa and Australia (Hoese and Larson 1994). The species like *Valenciennea longipinnis* (Lay & Bennett, 1839) and *Valenciennea muralis* (Valenciennes, 1837) were widely distributed in the eastern Indian Ocean. *Valenciennea parva* (Hoese & Larson, 1994), *Valenciennea strigata* (Broussonet, 1782) and *Valenciennea puellaris* (Tomiyama, 1956) were distributed in the Indo-Pacific from Red Sea to the Great Barrier Reef. The species *Valenciennea wardii* (Playfair, 1867) is rare and distributed in widely scattered localities in the Indian Ocean (Hoese and Larson 1994). Other species of this genus show narrow ranges. Distribution of *Valenciennea alleni* (Hoese & Larson, 1994) is restricted to the Australian coast whereas *Valenciennea bella* (Hoese & Larson, 1994) occurs along the coast of Japan and Philippines (Hoese and Larson 1994). *Valenciennea immaculata* (Ni, 1981) is distributed along the coast of Taiwan, Hongkong, the Philippines and Australia (Randall et al. 2004) and *Valenciennea limicola* (Hoese & Larson, 1994) occurs along the coast of Thailand and Fiji (Allen and Adrim 2003).

The nearest known record of *Valenciennea helsdingenii* is from the Maldives. The present report adds to our knowledge of species diversity of Gobiidae from the Bay of Bengal, and it assumes that the Bay of Bengal contains as many species as the entire western Indian Ocean. The long stretch of coral islands along the Gulf of Mannar and Andaman Nicobar Islands increases the chance of species abundance and richness in the Bay of Bengal. During recent years, great numbers of new fish species have been described and recorded from the east coast of India (Kannan et al. 2012, Joshi et al. 2012, Zacharia and Kannan 2012).

## Supplementary Material

XML Treatment for Valenciennea helsdingenii
